# Detailed Investigation of Downstream TLR Signaling in the Follicular Cells of Women with Endometriosis

**DOI:** 10.18502/jri.v21i4.4325

**Published:** 2020

**Authors:** Reza Jafari, Seyed Abdolvahab Taghavi, Elham Amirchaghmaghi, Reza Salman Yazdi, Leili Karimian, Mahnaz Ashrafi, Reza Aflatoonian

**Affiliations:** 1- School of Medicine, Shahroud University of Medical Sciences, Shahroud, Iran; 2- Fertility and Infertility Research Center, Hormozgan University of Medical Sciences, Bandarabbas, Iran; 3- Department of Endocrinology and Female Infertility, Reproductive Biomedicine Research Center, Royan Institute for Reproductive Biomedicine, ACECR, Tehran, Iran; 4- Department of Andrology, Reproductive Biomedicine Research Center, Royan Institute for Reproductive Biomedicine, ACECR, Tehran, Iran; 5- Department of Embryology, Reproductive Biomedicine Research Center, Royan Institute for Reproductive Biomedicine, ACECR, Tehran, Iran; 6- Department of Gynecology and Obstetrics, School of Medicine, Iran University of Medical Sciences, Tehran, Iran; 7- Department of Gynecology and Obstetrics, School of Medicine, Yasuj University of Medical Sciences, Yasuj, Iran

**Keywords:** Endometriosis, Follicular cells, Infertility, Inflammation, TLR

## Abstract

**Background:**

Inflammatory responses within the peritoneal cavity may result in endometrial dysfunction in women with endometriosis. The true causes of this disease remain poorly understood. It is hypothesized that downstream toll-like receptors (TLRs) inflammatory cytokines in response to pathogens may be associated with endometriosis. So, this study was aimed at evaluating the expression of TLRs signaling and endometriosis-associated inflammatory responses.

**Methods:**

Totally, 20 infertile endometriosis patients and 20 normal women undergoing controlled ovarian stimulation were enrolled. The cellular pellet and supernatant were obtained by centrifugation of follicular fluid (FF). Evaluation of TLRs and their signaling pathway gene expression was performed on cellular pellets using quantitative-PCR. The supernatant was used for determination of cytokine protein expression by ELISA. The results are expressed as mean±SEM and a p<0.05 was considered statistically significant.

**Results:**

Quantitative-PCR analysis suggested that TLR1, 5, 6, 7, 8, 10, MYD88, NF-ĸB, IL-10 and TGF-β genes expression significantly increased in patients compared to the control group (p<0.05). TLR3, 9, INF-β genes expression was significantly lower in endometriosis than control group (p<0.05). There was no significant difference in the expression of TLR2, TLR4, TIRAP, TRIF, TRAM, and IRF3 between two groups. Also, significant increase in the levels of IL-6, IL-8 and MIF protein in FF of endometriosis group was detected in comparison with normal women (p<0.05).

**Conclusion:**

The expression of TLR downstream signaling in the follicular cells can initiate inflammatory responses and changes in the FF cytokine profile which in turn may induce endometriosis and infertility disorder.

## Introduction

Endometriosis is a usual gynecologic disorder associated with infertility that affects reproductive-aged women (1). Endometriosis is an enigmatic condition in which endometrial glands or stromal cells grow in the extra-uterine locations such as fallopian tubes (2). There is no unifying theory that adequately explains the etiology and pathophysiology of endometriosis (3). However, genetic susceptibilities, hormonal or immunologic factors may be involved in the pathogenesis of the disease (4). In the last few decades, many more studies have illustrated the role of immune responses in the etiology and pathophysiology of endometriosis (5–7). Normally, the immune system can clear refluxed endometrial tissues. Therefore, the predisposition to implantation and growth of endometrial cells could be due to dysregulation of immune clearance mechanism (4).

Many studies have reported that engagement of toll-like receptors (TLRs) by gram-positive and gram-negative bacterias in the vaginal route can initiate inflammatory responses which leads to the promotion of infectious diseases such as endometritis (8, 9). TLRs play essential roles in the innate immune system by recognation of pathogen-associated molecular patterns (PAMPs) and damage-associated molecular patterns (DAMPs) (10). The expression of TLRs in the human female reproductive system has been established (11). By now, ten members of TLR family have been identified in human which are expressed on various immune and non-immune cells (12–14). TLR1, 2, 4, 5, 6 and TLR10 are expressed on the cell surface where as TLR3, 7, 8 and TLR9 are located in cellular endosome (10, 15). There are published studies that have reported TLRs 1–10 are expressed in the female reproductive tract (2, 16) and their expression level varies in different phases of menstrual cycles (17). *In vitro* studies have shown that ligation of TLRs on endometrial epithelial cell lines triggers the production of inflammatory cytokines including interleukin 6 (IL-6) and IL-8 (18). The expression of TLRs 2, 4, 8 and 9 in mouse granulosa and cumulus cells has also been reported (19). TLRs-ligand interaction activates downstream signaling pathways including adaptor molecules MYD88, nuclear factor kappa B (NF-kB) and interferon regulatory factor (IRF) 3 and 7 that enhance secretion of pro-inflammatory cytokines and chemokines such as interleukins 6, 8, 10 and macrophage migration inhibitory factor (MIF) (20). It was reported that the peritoneal fluid of women with endometriosis contained significantly elevated levels of the chemokines, monocyte chemoattractant protein-1 (MCP-1) and IL-8 (21). On the other hand, the proliferation of human endometrial stromal cells may be inhibited by interleukin-6 (22). Also, the severity of endometriosis is correlated with levels of IL-6 in peritoneal fluid (23). However, there is no strong evidence to support it and more detailed studies are needed to clarify all aspects of dysregulation of the immune system in the development and progress of endometriosis. The aim of the present study was investigation of changes in TLRs and their signaling pathway gene expression in the follicular cells of infertile women with endometriosis.

## Methods

### Study design:

This case-control study was approved by Ethics Committees of Royan Institute. Forty patients (20 infertile endometriosis patients and 20 normal women with male factor infertility as a control) who attended Reproductive Medicine Unit in the Royan Infertility Clinic, Tehran, Iran, for intracytoplasmic sperm injection (ICSI) treatment were the subjects of the study. An information sheet was offered to all women and informed written consent was obtained. The infertile women aged 20–35 undergoing ICSI treatment with the same standard long protocol and patients with stage III endometriosis in laparascopic assessment were selected. Stage III endometriosis was defined as the presence of endometriomas on the ovary (24). Patients with hyper-prolactinemia, poor ovarian response and history of ovarian surgery and infection were excluded. The anthropometric measurements were accomplished, based on body mass index [BMI, calculated as weight/(height)^2^ (*kg/m*^2^)]. The hormones such as LH, FSH, testosterone were also determined. All laboratory parameters were checked in the early follicular phase of the menstrual cycle.

### Protocol for controlled ovarian stimulation:

In this study, to minimize bias, all women were administrated a standard long protocol using GnRH-agonist (Superfact, Aventis, Germany). GnRH-a started around day 17–19 of the natural menstrual cycle as a pre-treatment. A daily dose of 0.5 *mg* was used subcutaneously when pituitary desensitization was confirmed (Endometrial thickness <5 *mm* and serum estradiol level <50 *pg/ml*), then the GnRH-a dose was reduced by one-half and ovarian stimulation was initiated. The ovarian stimulation started with a dose of 150–225 IU r-FSH (Gonal-F, Merck Serono, Switzerland) concerning the patient’s age and was continued until the day of ovulatory human chorionic gonadotropin (hCG) administration according to the ovarian response. When at least two follicles were greater than 18 *mm*, 10,000 *IU* urinary hCG (Choriomon, IBSA, Switzerland) was administered intramuscularly for ovulation induction and oocyte retrieval was performed 34–36 *hr* later (25).

### Sample collection:

FF aspiration was carried out with transvaginal ultrasound guidance aspirating the largest follicles (>18 *mm*) without flashing and blood contamination. The follicular fluid (FF) was transferred to a sterile Petri dish, then the oocytes were removed. The fluid was collected into a 15-*ml* conical tube and centrifuged at 300 *g* for 5 *min*.

### RNA isolation, cDNA synthesis and RT-PCR:

The supernatant was removed and 1ml of TRI reagent (Sigma, Pool, UK) was added to the cell pellet and homogenized for total RNA extraction following a standard protocol according to manufacturer’s instruction. Obtained total RNA, in both groups, was treated three times with DNase I (Fermentase, Germany) to remove genomic DNA contamination from the samples. The first-strand cDNA synthesis was performed using oligo (dT) primers and reverse transcription was done by SuperScript II (Fermentase). Negative controls were prepared without the addition of the enzyme (Non-reverse transcribed controls, RT controls). The RT-PCR was performed using the cDNA of each sample. Briefly, the amplification was persistent for 40 cycles under the following setting: 95°*C* for 30 *s*, 60–63°*C* (Table 1) for 30 *s*, and 72°*C* for 30 *s*. Β-actin and GAPDH were used as internal controls and their expressions were check-ed between two groups.

**Table 1.** List of primers

**Gene**	**Forward primer (5-3)**	**Reverse primer (3–5)**	**Annealing temperature (C)**	**Product size (*bp*)**
**TLR1**	GGGTCAGCTGGACTTCAGA	AAAATCCAAATGCAGGAACG	63	250
**TLR2**	TCGGAGTTCTCCCAGTTCTCT	TCCAGTGCTTCAACCCACAA	60	175
**TLR3**	GTATTGCCTGGTTTGTTAATTGG	AAGAGTTCAAAGGGGGCACT	60	156
**TLR4**	TGATGTCTGCCTCGCGCCTG	AACCACCTCCACGCAGGGCT	60	98
**TLR5**	CACCAAACCAGGGATGCTAT	CCTGTGTATTGATGGGCAAA	60	111
**TLR6**	GCCACCATGCTGGTGTTGGCT	CGCCGAGTCTGGGTCCACTG	60	101
**TLR7**	CCTTGAGGCCAACAACATCT	GTAGGGACGGCTGTGACATT	63	285
**TLR8**	CTTCGATACCTAAACCTCTCTAGCAC	AAGATCCAGCACCTTCAGATGA	60	90
**TLR9**	TTCCCTGTAGCTGCTGTCC	ACAGCCAGTTGCAGTTCACC	60	207
**TLR10**	TGCCCACCACAATCTCTTCCATGA	AGCAGCTCGAAGGTTTGCCCA	60	184
**MYD 88**	GTCTCCTCCACATCCTCCCT	TCCGCACGTTCAAGAACAGA	60	82
**TIRAP**	CCTGCTGAAGAAGCCCAAGA	GGTTGTCCTGTGAGGTAGGC	60	83
**TRIF**	GAAGGAACAGGACACCCGAG	TGAGTAGGCTGCGTTCAGTG	60	91
**TRAM**	TGAAGCCCTCAGAGTCCAGA	TCTGCCACATGGCATCTCAG	60	85
**NF-kB**	CTGGATCTGCTGGTGGACAG	CTGTGGCTAGATGCAAGGCT	60	82
**IRF 3**	CTTGGTGGAGGGCATGGATT	GTTGAGGTGGTGGGGAACAG	60	96
**IFN-β**	ACGCCGCATTGACCATCTAT	GTCTCATTCCAGCCAGTGCT	60	85
**IL-10**	AAGACCCAGACATCAAGGCG	AGGCATTCTTCACCTGCTCC	60	135
**TGF-β**	GGAGCAGCTGTCCAACATGA	GGGCACGGGTGTCCTTAAAT	60	146
**B-Actin**	CAAGATCATTGCTCCTCCTG	ATCCACATCTGCTGGAAGG	60	90
**GAPDH**	CTCATTTCCTGGTATGACAACGA	CTTCCTCTTGTGCTCTTGCT	60	122

### Quantitative real-time PCR (QPCR):

The Q-PCR was performed for evaluating myeloid differentiation primary response protein (MYD88), TIR domain containing adaptor protein (TIRAP), TIR-domain-containing adapter-inducing interferon-β (TRIF), TRIF-related adaptor molecule (TRAM), NF-ĸB, interferon regulatory factor 3 (IRF3), interferon-β (INF-β), IL-10 and transforming growth factor beta (TGF-β) with the CDNA prepared from the follicular cells pallet. The forward and reverse primers for each gene were designed by Primer-BLAST (https://www.ncbi.nlm.nih.gov/tools/primer-blast/) (Table 1). The qPCR reactions were carried out in triplicates using an ABI Prism 7300 Sequence Detector (Applied Biosystems, Foster, USA) in a total volume of 20 *μl* containing 250 *ng* of cDNA, 5 *pmol* of gene specific primers and SYBR green reagent (Applied Biosystems) with ROX dye as a passive control for signal intensity. The thermal cycle profile included 50 cycles at 95°*C* for 30 *s*, 60–63°*C* (Table 1) for 30 *s*, and 72°*C* for 30 *s* (26). Samples were run in triplicate. Melting curve analysis was used for th determination of the specificity of the PCR fragments. All melting curves produced one peak per PCR product. Standard curves were obtained using the logarithmic dilution series of total RNA. The efficiency of the RT-PCR was 98%. The qPCR data were analyzed using the comparative CT method (27) and fold change was calculated as FC–2^−∆∆CT^.

### Immunoassay:

The follicular fluid from each patient was centrifuged at 300 *g* for 5 *min*, then the supernatant was used to measure the concentration of IL-6, IL-8, and MIF by commercially enzyme-linked immunosorbent assay (ELISA) kits. Briefly, this technique uses a microwell plate coated with a monoclonal antibody specific for human IL-6 (eBioscience, minimal detection level <1 *pg/ml*), human IL-8/NAP-1 (eBioscience, minimal detection level <5 *pg/ml*), and human MIF (Glory Science, minimal detection=3 *mg/ml*). The preparation was added to each well and incubated at room temperature for 1 *hr*. Then, plates were washed three times using phosphate-buffered saline (PBS). The biotin-conjugated anti-human IL-6, IL-8, and MIF monoclonal antibody was added to each plate. The plates were washed again and streptavidin-HRP and tetramethyl-benzidine as a substrate was added to each well. Optical density (OD) was measured by ELISA reader at 450 *nm*. The concentration of each cytokine was determined by comparing the optical density of samples.

### Statistics:

The results were expressed as mean± SEM. Statistical analysis was performed by using a t-test using SPSS 22 software. The p<0.05 was considered significant. For real-time data, 2^–∆∆Ct^ was calculated for each gene, then the data was analyzed using the GraphPad PISM 8 software.

### Ethical consideration:

All procedures were approved by the Ethics Committee of Royan Institute and informed consent was obtained from each patient before the sample collection.

## Results

The demographic and clinical characteristics of patients are presented in table 2. The number of mature oocyte is significantly lower in endometriosis samples compared to the control group. There is no significant difference in other items between the two groups.

**Table 2.** Demographic and clinical characteristics of patients

**Variable**	**Endometriosis (n=20)**	**Control (n=20)**	**p**
**Age (Years)**	29.78±4.52	30.75±3.89	0.48
**BMI (*kg/m*^2^)**	24.75±3.19	25.20±4.19	0.70
**LH (*mU/ml*)**	3.41±1.65	5.02±3.39	0.07
**FSH (*mU/ml*)**	6.86±2.80	8.55±5.73	0.23
**LH/FSH ratio**	0.55±0.30	0.64±0.33	0.40
**Testosterone (*ng/ml*)**	1.35±0.44	1.31±0.54	0.87
**No. of mature oocytes**	5.35±4.5	10.1±1.59	<0.001

Presented as mean±SD and compared by t-test

BMI: Body Mass Index

**Figure 1.**
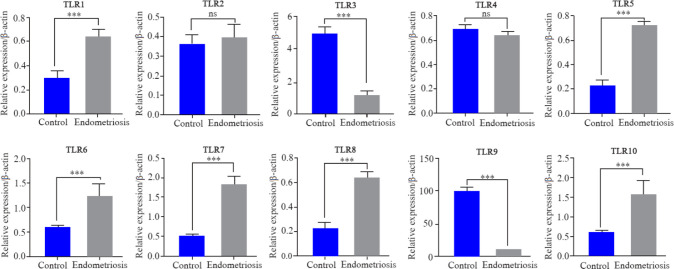
QPCR was used to quantify the expression of TLRs mRNA in endometriosis and control groups. Data are presented as mean±SEM of normalized expression values against endogenous controls (B-actin and GAPDH mRNA) in endometriosis and control group. TLR1, 5, 6, 7, 8 and TLR10 showed a significantly higher expression in endometriosis patients compared with the normal women. TLR2 and TLR4 showed no significant difference in both groups. TLR3, 9 showed significantly lower expressions in endometriosis patients compared with the control. Data were analyzed by t-test. The level of significance was set at p<0.05.

**Figure 2.**
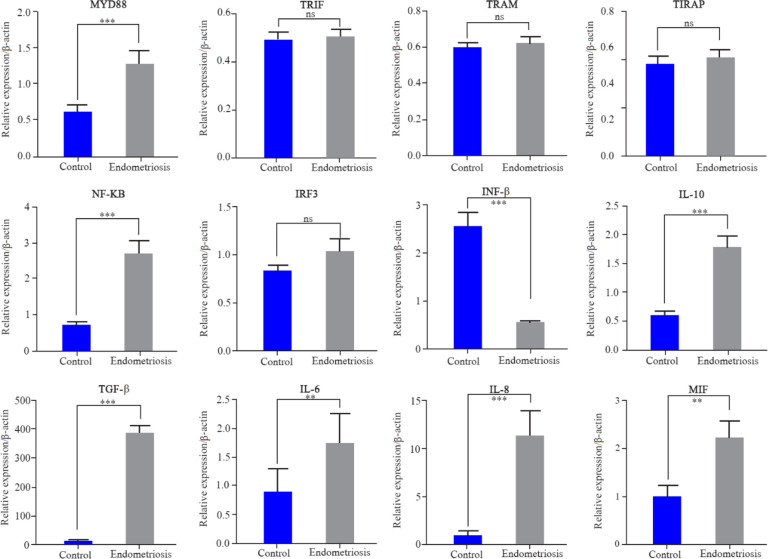
Q-PCR was used to quantify the expression of MYD88, TIRAP, TRIF, TRAM, NF-ĸB, IRF3, INF-β, IL-10 and TGF-β genes in granolosa cells in endometriosis and control groups. Data are presented as mean±SEM of normalized expression values against endogenous controls (B-actin and GAPDH mRNA) in POR and control. MYD88, NF-ĸB, IL-10 and TGF-β showed a significantly higher expression in endometriosis patients compared with the control. The expression of TIRAP, TRIF, TRAM, and IRF3 revealed no significant difference in endometriosis patients compared to the control group. INF-β showed significantly lower expression in endometriosis patients compared with the control group. Data were analyzed by t-test. The level of significance was set at p<0.05

The quantitative expression profiles of TLR1–10 genes in follicular cells in both groups are shown in figure 1. TLR1, 5, 6, 7, 8 and TLR10 showed a significantly higher expression in endometriosis patients compared to the control (p<0.05). The fold change and exact p-value of each gene are shown in table 3. There was no significant difference in the expression of TLR2 and 4 in both groups. TLR3, 9 showed significantly lower expressions in endometriosis patients compared with the control (p<0.05). The quantitative expression profile of MYD88, TIRAP, TRIF, TRAM, NF-ĸB, IRF3, INF-β, IL-10 and TGF-β genes in follicular cells in both groups are shown in figure 2. MYD88, NF-ĸB, IL-10, and TGF-β showed a significantly higher expression in endometriosis patients compared to the control (p<0.05). The expression of TIRAP, TRIF, TRAM, and IRF3 revealed no significant difference in endometriosis patients compared to the control group. The interferon-β (INF-β) showed significantly lower expression in endometriosis patients compared with the control (p<0.05).

**Table 3.** The fold change and p-value of each gene

**Gene**	**Fold change**	**p-value (<0.05)**
**TLR1**	5.142	0.0009
**TLR2**	1.13	0.2711
**TLR3**	12.16	0.0001
**TLR4**	1.96	0.5239
**TLR5**	3.23	0.021
**TLR6**	5.95	0.0011
**TLR7**	7.07	0.0003
**TLR8**	8.33	0.0003
**TLR9**	12.931	<0.0001
**TLR10**	11.89	0.00001
**MYD88**	10.03	0.0028
**TRIF**	1.34	0.6701
**TRAM**	2.07	0.2839
**TRAP**	0.94	0.6086
**NF-kB**	13.80	0.0055
**IRF3**	1.78	0.5741
**INF-β**	6.02	0.0012
**IL-10**	5.12	0.0052
**TGF- β**	9.77	0.0021
**IL-6**	14.75	<0.00001
**IL-8**	20.14	<0.00001
**MIF**	18.98	<0.00001

**Figure 3.**
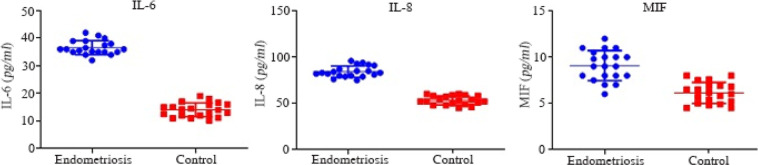
IL-6, IL-8 and MIF protein concentration obtained by ELISA in FF of endometriosis and control groups. IL-6, IL-8, and MIF showed a significantly higher expression in endometriosis cases compared with the control. Data were analyzed by t-test. The level of significance was set at p<0.05

The quantitative analysis of IL-6, IL-8, and MIF concentration in FF is shown in figure 3. IL-6, IL-8, and MIF significantly increased in endometriosis cases as compared with the control (p<0.05).

## Discussion

Our study was performed to expand our knowledge of the molecular aspects of immune responses in patients with endometriosis. Both anti-inflammatory cytokines, TGF-β and IL-10 and the inflammatory mediators, IL-8, IL-6, MIF, and interferon were investigated as well.

It is hypothesized that dysregulation of immune responses has been involved in the pathophysiology of endometriosis-associated infertility (28). Accordingly, infertile women with endometriosis have a significantly higher expression of TLR1, 5, 6, 7, 8, 10 and significantly lower expression of TLR3, 9 when compared with normal women. However, the expression of TLR2 and 4 was not significantly different between both groups. A significant increase of TLR4 mRNA expression was reported in the ectopic endometriotic lesions compared to corresponding eutopic tissues by Allhorn et al. (29). However, our results indicated that expression of TLR4 in follicular cells of women with endometriosis was not significant compared to the control.

Emerging evidence reported that some of the pro-inflammatory cytokines and chemokines are increasing not only in the endometrium but also in the peritoneal fluid and follicular fluid of women and animals with endometriosis (30–32). Also, many studies have demonstrated pelvic inflammation that is triggered primarily by bacterial endotoxin (Lipopolysacccharide) and is mediated by toll-like receptors and their involvement in the development of pelvic endometriosis has been shown. Our findings suggested that simultaneously with TLR expression in the follicular cells, inflammatory and anti-inflammatory mediators such as IL-8, IL-6, and MIF also increased in follicular fluid. Our findings demonstrate the elevated MIF protein production in these patients which is consistent with previous studies that have shown elevated MIF in peritoneal fluid (33) and peripheral blood of women with endometriosis (34). Increased levels of MIF have adverse effects on capacitation and sperm motility (35). Moreover, MIF binding to CD74 and upregulated IL-8 lead to regulation of inflammatory and immune responses (36, 37). Surprisingly, a significant expression of anti-inflammatory cytokine, TGF- β was seen in follicular cells of women with endometriosis in comparison with control group. It has been reported that, cell adhesiveness, migration, colonization, invasiveness, and development of endometriosis can be increased by high level of TGF-β (38). The interaction between macrophages and endometrial stromal cells (EMSs) may stimulate the secretion of TGF-β and IL-10 that leads to downregulation of NK cells cytotoxicity and may promote the development of endometrial stripe (EMS) via the immune escape of ectopic fragments (39). Accordingly, a significant increase of TGF-β and IL-10 in FF was detected by immunoassay analysis. Also, TGF-β is believed to play a major role in the etiology of peritoneal endometriosis and this may provide an environment favorable to lesion formation (40, 41). The interaction between activated macrophages or maybe follicular cells (TLR stimulation via microbial products leads to macrophages activation) and other stromal cells in women with endometriosis downregulates activation of immune cells, possibly by stimulating the secretion of IL-10 and TGF-β, and may further trigger the immune escape of ectopic fragments and promote the occurrence and the development of endometriosis in women (39, 40). TGF-β and IL-10 are secreted by many cell types, including macrophages. Inflammatory stimuli (IL-2, IL-10, TGF-β), that activate macrophages and other cells, such as follicular cells enhance the release of active TGF-β and IL-10. Moreover, TGF-β signalling is a well-studied pathway involved in follicular development and ovulation (42). Generally, our findings disclosed that increased expression of TLRs molecules in folicular cells leads to recruitment and increase of different adaptor molecules such as MyD88, TIRAP, TRAM and TRIF which results in activation of transcription factors including NF-KB and IRFs which in turn induces production of pro-inflammatory cytokines in follicular fluid. Therefore, more detailed investigations are needed to clarify the role of inflammatory or anti-inflammatory responses in the development of endometriosis lesions.

## Conclusion

Our results suggest that the lack of clearance of microbial agents may lead to the identification of pathogen-related structures by TLRs which in turn triggers the adapter and downstream signaling molecules such as NF-KB, MYD-88 and TRIF. As a result, TLRs signaling pathway induces production of inflammatory cytokines and chemokines especially IL-6, IL-8 and MIF in follicular tissues. Increased inflammatory responses and changes in the FF cytokine profile may causes or worsen endometriosis and infertility.
